# A Tryptophan Metabolite of the Microbiota Improves Neovascularization in Diabetic Limb Ischemia

**DOI:** 10.3389/fcvm.2022.910323

**Published:** 2022-06-02

**Authors:** Xiurui Ma, Jinjing Yang, Guanrui Yang, Lei Li, Xiaojun Hao, Guoqin Wang, Jian An, Fei Wang

**Affiliations:** Department of Cardiology, Shanxi Cardiovascular Hospital, Taiyuan, China

**Keywords:** diabetes mellitus, limb ischemia, indole-3-aldehyde, mitochondrial dysfunction, oxidative stress, apoptosis

## Abstract

Diabetes mellitus (DM) is accompanied by a series of macrovascular and microvascular injuries. Critical limb ischemia is the most severe manifestation of peripheral artery disease (PAD) caused by DM and is almost incurable. Therapeutic modulation of angiogenesis holds promise for the prevention of limb ischemia in diabetic patients with PAD. However, no small-molecule drugs are capable of promoting diabetic angiogenesis. An endogenous tryptophan metabolite, indole-3-aldehyde (3-IAld), has been found to have proangiogenic activity in endothelial cells. Nevertheless, the role of 3-IAld in diabetic angiogenesis remains unknown. Here, we found that 3-IAld ameliorated high glucose-induced mitochondrial dysfunction, decreasing oxidative stress and apoptosis and thus improving neovascularization.

## Introduction

Diabetes mellitus (DM) continues to be an epidemic affecting people worldwide. Globally, nearly 500 million people live with diabetes, and the number is expected to increase by more than 50 percent in the next 25 years (1). DM is a major risk factor for cardiovascular disease, and prolonged diabetes leads to an increased incidence of peripheral artery disease (PAD; 2, 3). Critical limb ischemia (CLI) is a common pathological manifestation of PAD characterized by ischemic rest pain, foot ulcers and gangrene, with a high risk of lower limb amputation and poor prognosis (4). Current therapies, such as surgical revascularization and medication, have limited benefits for CLI patients.

Microbiota-dependent tryptophan catabolites are abundantly produced within the gastrointestinal tract and are known to exert profound effects on host physiology, including the maintenance of epithelial barrier function, endothelial cell (EC) angiogenesis and immune homeostasis (5, 6). Several of the indole derivatives produced by the gut microbiota, including indole-3-aldehyde (3-IAld), are suggested ligands for the aryl hydrocarbon receptor (AHR), a xenobiotic response receptor (7). Notably, endogenous tryptophan metabolism produces several metabolites that are AHR ligands that have been implicated in autoimmunity and cancer (8, 9). Recent analyses suggest that 3-IAld has anti-inflammatory activity and proangiogenic effects, which apparently may involve mechanisms not dependent on AHR (5).

Angiogenesis is critical for the repair of ischemic wounds and tissues (10). Reactive oxygen species (ROS) are a heterogeneous group of highly reactive ions and molecules derived from molecular oxygen (O_2_; 11), and various lines of evidence support the fact that the biological roles of ROS are complex and paradoxical (12, 13). Excessive production of ROS can cause oxidative stress, which may damage cellular lipids, proteins, and DNA (11, 14), thus promoting apoptosis and decreasing cell proliferation, migration and angiogenesis. Therefore, balancing the ROS level is important for regulating cell proliferation, apoptosis, migration, and angiogenesis. Mitochondrial damage is an important source of ROS in most mammalian cells (15). The production of ROS leads to mitochondrial damage in a series of pathological processes and plays an important role in redox signaling (16, 17).

It has been shown that 3-IAId promotes angiogenesis in ECs. Nevertheless, very little is known about the function of tryptophan metabolites, in particular 3-IAId, in diabetic neovascularization. We therefore hypothesized that 3-IAId plays a protective role in diabetic angiogenesis. In this study, we used STZ-induced mice and HG (high glucose)-treated ECs to evaluate the role of 3-IAId in modulating diabetic angiogenesis.

## Materials and Methods

### Animals

Male C57BL/6 mice aged 8 weeks were purchased from Shanghai JieSiJie Laboratory Animal Center (Shanghai, China). As previously described, mice were kept at room temperature on a 12/12 light/dark cycle with free access to water and standard laboratory mouse diets. The animal experiment was approved by the Animal Protection and Utilization Committee of Shanxi Cardiovascular Hospital. Mice were intraperitoneally injected with 50 mg/kg streptozotocin (STZ, Sigma–Aldrich) on 5 consecutive days to induce diabetic hyperglycemia (18). At the 3rd month of DM induction, left hindlimb ischemia (HLI) was induced by femoral artery ligation (19). 3-IAId (Sigma) was dissolved in DMSO and delivered by oral gavage at a dose of 150 mg/kg/day in a final mixture of DMSO (20%), PEG 400 (40%), and citric acid (2%). 3-IAId treatment was started on the 5th day after STZ injection and continued throughout the experiment.

### Femoral Artery Ligation

Mice were anesthetized by intraperitoneal injection of 1% pentobarbital sodium. When the mice were unconscious and the muscles were relaxed, the mice were placed supine and fixed on the operative table. Alcohol (75%) was used for skin disinfection. Hair removal cream was used to remove the hair of both lower limbs. The skin was cut near the exposed oval to expose the femoral artery. Then, the accompanying veins and nerves were separated. The femoral artery and vein were ligated with 4–0 silk thread. Then, ophthalmic scissors were used to cut the artery below the ligation line, and the skin was sutured with 6–0 thread.

### Laser Doppler Perfusion Imaging

Blood flow restoration was assessed by laser Doppler perfusion imaging (PeriScan PIM 3 system, Perimed, Sweden). Briefly, mice were anesthetized with isoflurane and placed on a heating pad at 37°C. Limb perfusion was detected on Days 1, 3, 7, 14 and 21 after ligation. Blood perfusion was quantified as a percentage of blood flow in the ischemic versus non-ischemic hind limb.

### Wound Healing Assay

First, a straight line was drawn on the back of the plate to mark the observation of each area under a mirror. Human umbilical vein endothelial cells (HUVECs) were seeded onto 24-well plates in complete medium until they were 95% confluent. HUVECs were pretreated with or without HG for 24 h. Then, the complete medium was replaced with serum-free medium. Confluent cells were wounded by making a perpendicular scratch with a 200 μL pipette tip. Then, the supernatant was discarded, and fresh serum-free medium was added. The edges of the wound were measured at 0 and 24 h after scratching at the same points according to the markers. ImageJ software was used to calculate the migration distance.

### Tube Formation Assay

Human umbilical vein endothelial cells were seeded onto 6-well plates in complete medium and were pretreated with or without HG or 3-IAId for 36 h. Twenty-four-well culture plates were coated with 200 μL of Matrigel (BD Biosciences, San Diego, CA, United States). HUVECs were digested with trypsin, and 1 × 10^5^ cells/well were seeded onto Matrigel. The cells were incubated for an additional 12 h in the absence or presence of high glucose (25 mM; Sigma–Aldrich; 20) or 3-IAId (0.5 mM; Sigma–Aldrich; 5). Images were captured by microscopy (Olympus), and the lengths of the capillary networks were quantified using ImageJ software.

### Aortic Ring Sprouting Assay

Aortas were isolated from the mice under sterile conditions and flushed with saline to remove residual blood. Then, aortic tissues were cut into 1 mm × 1 mm pieces. Twenty-four-well culture plates were coated with 200 μL of Matrigel (BD Biosciences, San Diego, CA, United States). The aortic tissue was plated on the surface of the Matrigel, followed by addition of a drop of matrix on the aortic tissue to sandwich the aorta in the middle of the Matrigel. Then, complete culture medium (ECGF, 5 U/ml heparin, 100 U/ml penicillin, 100 μg/ml streptomycin, and 20% fetal calf serum) was added to the 24-well plate. 5 days later, sprouting of the vascular tissue was observed by microscopy (Olympus).

### Immunofluorescence Staining

Paraffin-embedded sections (6 μm) of ischemic hind limb skeletal muscle from each group were subjected to Immunofluorescence (IF) staining. The cells were rinsed with PBS 3 times for 5 min each and then blocked with 10% goat serum at 37°C for 1 h. Next, the cells were incubated with an anti-CD31 (AF3628, R&D) antibody at 4°C overnight. Sections were washed three times with PBS and labeled with a secondary antibody (Invitrogen) at 37°C for 1 h. DAPI (Invitrogen) was used to stain the nuclei. Sections were imaged by confocal fluorescence microscopy (Leica, Germany).

### Intracellular and Mitochondrial Reactive Oxygen Species Measurement

The production of ROS was evaluated by analyzing the fluorescence intensity by dihydroethidium (DHE; Invitrogen), DCFDA (Abcam), and MitoSOX (Invitrogen) staining. In brief, frozen musculus gastrocnemius sections were stained with 5 μM DHE at 37°C for 30 min, and then fluorescence intensity was detected by confocal microscopy. HUVECs were stimulated with high glucose (25 mM; Sigma–Aldrich; 20) or 3-IAId (0.5 mM; Sigma–Aldrich; 5) for 24 h, subjected to DCFDA (20 μM), or mitoSOX (5 μM) staining at 37°C for 30 min and then assessed by fluorescence microscopy.

### TUNEL Staining

TUNEL staining was performed to detect cellular apoptosis in the gastrocnemius sections after femoral artery ligation according to the TUNEL staining kit manufacturer’s instructions (Abcam, Cambridge; United Kingdom; cat no. ab66110). In brief, tissue samples were fixed in 4% paraformaldehyde, embedded in paraffin, and cut into 5 mm transverse sections. Paraffin sections of the aorta were stained with an *in situ* BrdU-Red DNA Fragmentation (TUNEL) Assay Kit (ab66110, Abcam). 4′,6′-Diamidino-2-phenylindole (DAPI) was used for nuclear counterstaining. HUVECs were stimulated with high glucose (25 mM; Sigma–Aldrich; 20) or 3-IAId (0.5 mM; Sigma–Aldrich; 5) for 24 h and then subjected to TUNEL staining at 37°C for 30 min. The number of TUNEL-positive nuclei was analyzed using ImageJ software (NIH, Bethesda, MD, United States). The TUNEL-positive cell number per field for each sample was the average of 6 random fields.

### Metabolic Analysis

To characterize metabolic changes in HUVECs from each group, 20,000 cells/well were plated on gelatin-coated 96-well plates for Seahorse analysis 36 h before detection in a 1:1 EGM2 (Promo Cell) and fully supplemented DMEM mixture. A Seahorse XF96 analyzer was used for analysis according to the Agilent protocol (Mito Stress Kit). The metabolic function parameters shown in bars were calculated according to the Agilent protocol: basal respiration: [(final rate before oligomycin treatment) – (minimum rate after rotenone/antimycin A injection) = (non-mitochondrial oxygen consumption)]; ATP production: [(final rate before oligomycin injection) – (minimum rate after oligomycin injection)]; and spare respiratory capacity: [(measurement of maximum rate after injection of FCCP) – (non-mitochondrial respiration)] = [(maximum respiration) – (basal respiration)].

### Statistical Analysis

Data were analyzed in GraphPad Prism 9.0 (GraphPad Software, San Diego, CA, United States). Non-normally distributed data were logarithmically converted prior to analysis and represented in the median and quartile ranges. Data are expressed as the mean ± SEM. Statistical significance was assessed by two-way ANOVA with Bonferroni correction for multiple comparisons. *p* < 0.05 was considered statistically significant.

## Results

### Indole-3-Aldehyde Improves Blood Perfusion and Ischemic Angiogenesis in Diabetic Hindlimb Ischemia

**FIGURE 1:**
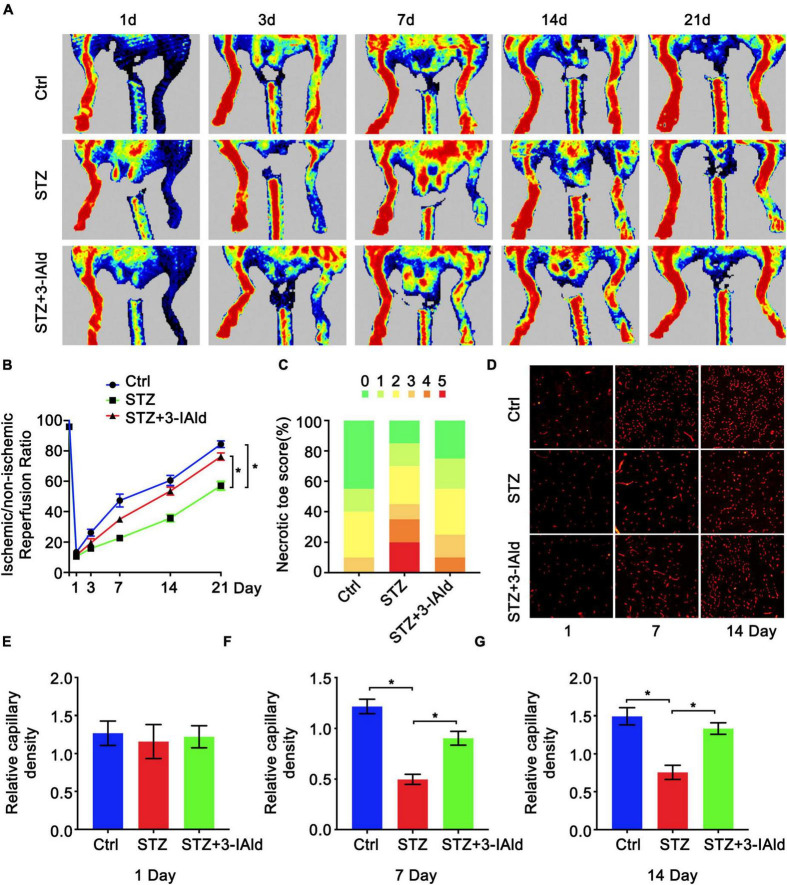
3-IAld improves blood perfusion and ischemic angiogenesis in diabetic HLI. **(A)** Original laser Doppler perfusion images (LDPIs) displaying hindlimb perfusion in each group at 1, 3, 7, 14, and 21 days after ligation of the femoral artery. **(B)** Perfusion recovery in mice of the control, STZ and STZ + 3-IAId groups at 1, 3, 7, 14, and 21 days after surgery. *n* = 10 for each group. **(C)** Necrotic toe score determined at Day 21 after ischemic injury. *n* = 7 for each group. **(D)** Representative immunofluorescence images showing CD31 density in gastrocnemius muscle at 1 and 14 days after artery ligation. **(E–G)** Capillary density was evaluated as the ratio of capillary number per field; *n* = 6 for each group. Data are presented as the mean ± SEM. *P < 0.05.

To determine the protective role of 3-IAId against diabetic ischemia, a diabetic model was established by STZ induction, and HLI was induced by femoral artery ligation. Mice in the experimental group were given 3-IAId (150 mg/kg/day) by oral gavage over 21 days. Blood perfusion of the hind limb footpad was evaluated by laser Doppler perfusion imaging technology on Days 1, 3, 7, 14 and 21 after surgery. The results showed that diabetes impaired hind limb blood perfusion recovery in both saline- and 3-IAId-treated mice in a time-dependent manner (Figures 1A,B). However, 3-IAId administration in STZ mice promoted greater flow recovery at Days 3–21 after HLI surgery compared with that in diabetic Ctrl mice (Figures 1A,B). Furthermore, on Day 21 after femoral artery ligation, the number of necrotic toes in each mouse was counted. Then, mice with different numbers of necrotic toes were classified and quantified, yielding necrotic toe scores. The necrotic degree reflected by the necrotic toe number was considerably lower in 3-IAId and STZ-treated mice than in the corresponding control mice (Figure 1C). Consistent with these results, 3-IAId-treated mice with diabetes also had increased capillary density in the gastrocnemius measured 7 and 14 days after HLI compared with that in diabetic Ctrl mice (Figures 1D–G). Thus, we conclude that 3-IAId improved blood flow recovery and ameliorated necrosis.

### Indole-3-Aldehyde Protects Endothelial Cells From High Glucose-Induced Angiogenic Dysfunction

**FIGURE 2:**
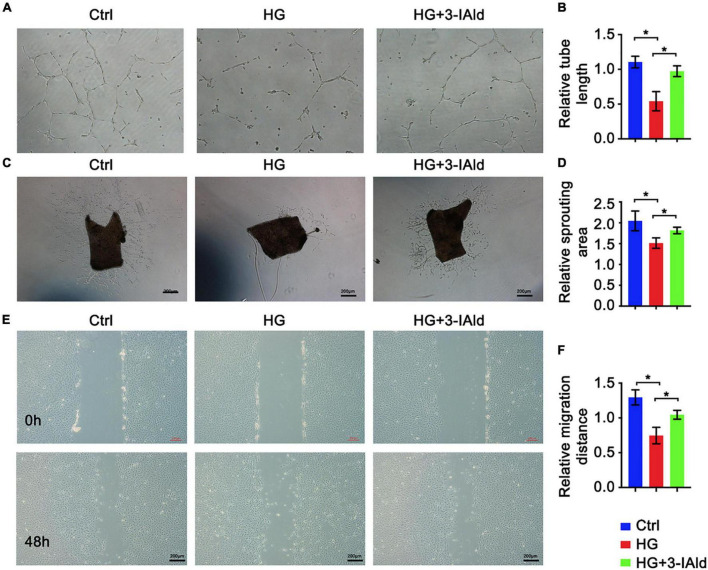
3-IAld protects ECs from HG-induced angiogenic dysfunction. **(A)** Original micrograph of tube formation in HUVECs in the control, HG and HG + 3-IAId groups; scale bar: 100 μm. **(B)** Quantitative analysis of relative tube length; *n* = 6 for each group. **(C)** Original micrograph of sprouts grown from aortic rings in the control, HG and HG + 3-IAId groups; scale bar: 200 μm. **(D)** Quantitative analysis of the relative sprouting area; *n* = 6 for each group. **(E)** Original micrographs of the wound healing assay; scale bar: 200 μm. **(F)** Quantitative analysis of wound healing assay in control, HG and HG + 3-IAId groups in HUVECs; *n* = 6 for each group. Data are presented as the mean ± SEM. *P < 0.05.

Endothelial cell migration, sprouting and tube formation are critical processes for angiogenesis, in which the formation of capillary-like tubes by ECs is a crucial step for blood flow recovery *in vivo*. We assessed the contribution of 3-IAId to EC biological functions *in vitro*. Obviously, 3-IAId treatment significantly reversed HG-induced damage to EC tube formation, EC migration and angiogenic sprouting from the aortic vascular wall (Figures 2A–F). Taken together, our findings indicate that 3-IAId regulates EC biological functions essential for angiogenesis, including enhanced migration, sprouting and tube formation.

### Indole-3-Aldehyde Alleviates Oxidative Stress and Apoptosis in Diabetic Hindlimb Ischemia

**FIGURE 3:**
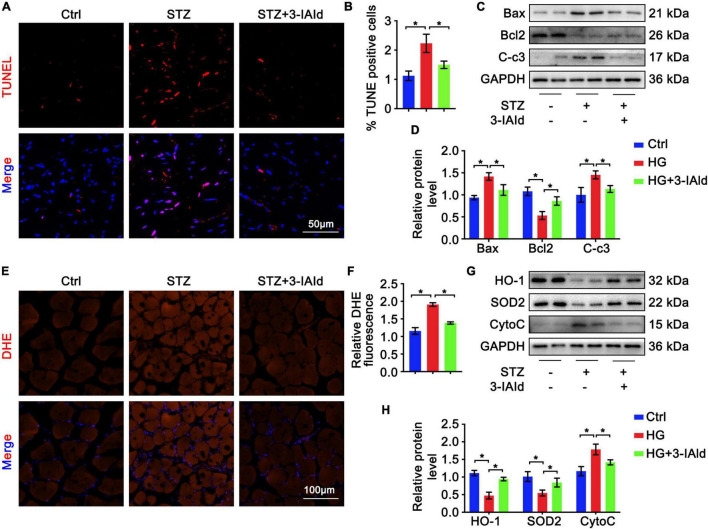
3-IAld alleviates oxidative stress and apoptosis in diabetic HLI. **(A)** TUNEL staining of diabetic mice in the control, STZ and STZ + 3-IAId groups at 7 days after ligation; scale bar: 50 μm. **(B)** Quantification of TUNEL staining; *n=6* for each group. **(C)** Western blot analysis of apoptosis-related molecules in the diabetic mice in the control, STZ and STZ + 3-IAId groups at 7 days after ligation. **(D)** Quantification of relative protein levels; *n* = 6 for each group. **(E)** DHE staining of diabetic mice in the control, STZ and STZ + 3-IAId groups at 7 days after ligation; scale bar: 100 μm. **(F)** Quantification of DHE staining; *n=6* for each group. **(G)** Western blot analysis of oxidative stress-related molecules in the diabetic mice in the control, STZ and STZ + 3-IAId groups at 7 days after ligation. **(H)** Quantification of relative protein levels; *n* = 6 for each group. Data are presented as the mean ± SEM. *P < 0.05.

Oxidative stress and apoptosis are critical in angiogenesis. Therefore, gastrocnemius sections from each group were subjected to TUNEL staining, and the results showed that STZ treatment exacerbated whereas 3-IAId administration attenuated cell apoptosis (Figures 3A,B). Moreover, immunoblot analysis of anti-apoptosis (Bcl2) and pro-apoptosis (Caspase3 and Bax) molecules revealed that the apoptosis exacerbation induced by STZ was rescued by 3-IAId supplementation (Figures 3C,D). Additionally, DHE staining of gastrocnemius muscle sections of each group showed that STZ treatment increased cell ROS production (Figures 3E,F), while 3-IAId administration reduced cell oxidative stress, which was further proven by immunoblot analysis (Figures 3G,H). Collectively, our data suggest that 3-IAId alleviates STZ-induced ROS production and thus blunts cell apoptosis.

### Indole-3-Aldehyde Protects Endothelial Cells From High Glucose-Induced Oxidative Stress and Apoptosis

**FIGURE 4:**
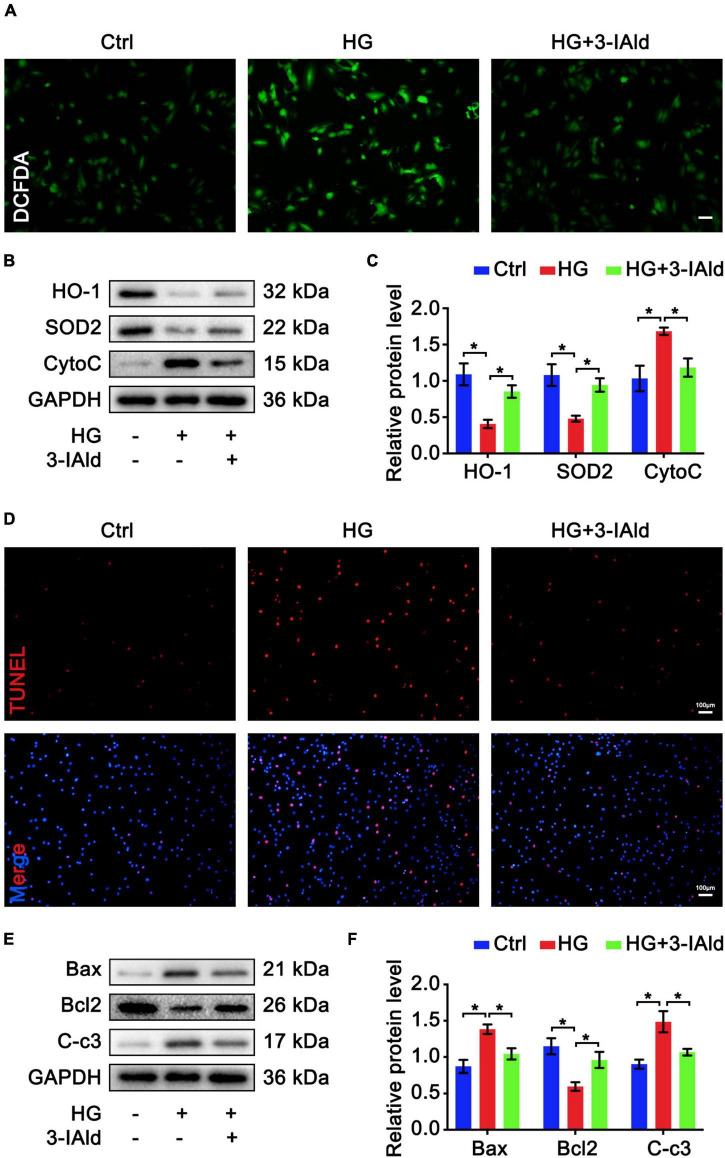
3-IAld protects ECs from HG-induced oxidative stress and apoptosis. **(A)** DCFDA staining in HUVECs in the control, HG and HG + 3-IAId groups; scale bar: 50 μm. **(B)** Western blot analysis of oxidative stress-related molecules in HUVECs in the control, HG and HG + 3-IAId groups. **(C)** Quantification of relative protein levels; *n* = 6 for each group. **(D)** TUNEL staining in HUVECs in the control, HG and HG + 3-IAId groups; scale bar: 100 μm. **(E)** Western blot analysis of apoptosis-related molecules in HUVECs in the control, HG and HG + 3-IAId groups. **(F)** Quantification of relative protein levels; *n* = 6 for each group. Data are presented as the mean ± SEM. *P < 0.05.

Furthermore, we validated the antioxidant and anti-apoptotic effects of 3-IAId *ex vivo*. Cultured ECs were treated with HG for oxidative stress and apoptosis induction with or without 3-IAId administration. ECs were subjected to DCFDA staining for ROS detection. In accordance with previous results, 3-IAId was able to attenuate the exacerbated oxidative stress induced by HG stimulation (Figure 4A). Immunoblot analysis of oxidant (Cytochrome c, CytoC) and antioxidant (HO-1 and SOD2) molecules revealed that 3-IAId treatment reduced EC oxidative stress (Figures 4B,C). TUNEL staining and immunoblot analysis of EC apoptosis also led to the same conclusion as that reached based on the *in vivo* results (Figures 4D–F). These findings denote the protective role of 3-IAId in EC oxidative stress and apoptosis.

### Indole-3-Aldehyde Attenuates High Glucose-Induced Mitochondrial Dysfunction in Endothelial Cells

**FIGURE 5:**
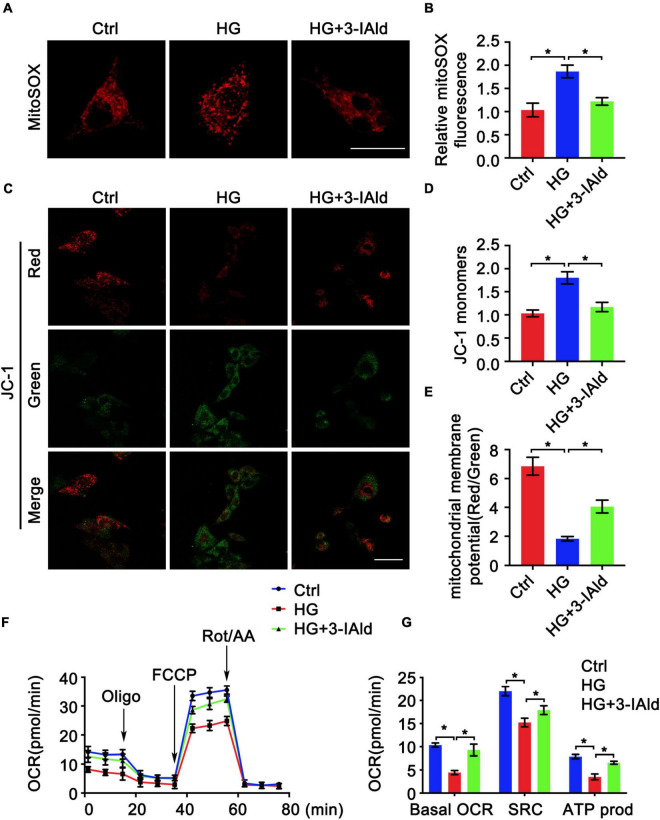
3-IAld attenuates HG-induced mitochondrial dysfunction in ECs. **(A)** MitoSOX staining in HUVECs in the control, HG and HG + 3-IAId groups, scale bar: 25 μm. **(B)** Quantification of relative MitoSOX fluorescence; *n* = 6 for each group. **(C)** JC-1 staining in HUVECs in the control, HG and HG + 3-IAId groups; scale bar: 50 μm. **(D,E)** Quantification of relative JC-1 monomers and mitochondrial member potential; *n* = 6 for each group. **(F)** Oxygen consumption rates (OCRs) in HUVECs in the control, HG and HG + 3-IAId groups before and after sequential injection of oligomycin, FCCP and a mixture of rotenone/antimycin A, as determined by a Seahorse XF96 Analyzer. **(G)** Corresponding calculated parameters of mitochondrial respiration; *n* = 6 for each group. Data are presented as the mean ± SEM. *P < 0.05.

Mitochondrial dysfunction promotes ROS production, which exacerbates mitochondrial damage (15, 16). We therefore investigated whether 3-IAId would affect mitochondrial function. Measurement of mitochondrial ROS by MitoSOX staining revealed that 3-IAId significantly alleviated HG-induced mitochondrial ROS production (Figures 5A,B). The mitochondrial membrane potential is the central bioenergetic parameter that controls respiratory rate, ATP synthesis and the generation of ROS (21). We further assessed the mitochondrial membrane potential by JC-1 staining. As expected, 3-IAId also played a role in the maintenance of the mitochondrial membrane potential (Figures 5C–E). For detailed metabolic characterization of ECs in each group, the respiratory capacity of the ECs was assessed using a Seahorse extracellular flux (XF) analyzer. As expected, the oxygen consumption rate (OCR) was drastically reduced in HG-treated ECs, with a significant reduction in the calculated basal and spare respiratory capacities (Figures 5F,G). Furthermore, the proportion of basal respiration used to drive ATP production was also dramatically reduced (Figures 5F,G). 3-IAId supplementation significantly improved cellular respiration in the mitochondria, as demonstrated by an increased OCR. Collectively, our findings suggest the protective role of 3-IAId in EC mitochondrial dysfunction.

## Discussion

The salient findings of our study confirmed for the first time that 3-IAId supplementation improved diabetic angiogenesis and limb perfusion. This finding was supported by data from a limb ischemia model in STZ-induced diabetic mice and *in vitro* experiments in HG-induced HUVECs. Our data further revealed that 3-IAId improved EC migration, tube formation and sprouting. 3-IAId supplementation improved angiogenesis, in part possibly because it reduced apoptosis and oxidative stress. Moreover, we confirmed that the beneficial effects of 3-IAId on EC apoptosis and oxidative stress were due to the improvement of mitochondrial function. This study described both the critical role and potential mechanisms of 3-IAId in diabetic angiogenesis.

Arterial occlusion leads to impaired angiogenesis in response to ischemia, contributing to serious clinical complications in diabetic patients with PAD or CAD (22, 23). The mechanisms that explain impaired angiogenic responses after diabetic ischemia vary, such as vascular degeneration characterized by either endothelial or vascular smooth muscle cell dysfunction (24), the reduced release or functional defects of endothelial progenitor cells in bone marrow (25), or the dysregulation of vascular growth factor pathways (26). Although various factors have been reported to be responsible for impaired angiogenesis in diabetic ischemia, the microbial metabolites involved in diabetic angiogenesis remain to be explored.

Recently, the microbiome affecting health and the pathogenesis of disease has received extensive attention. One of the modes by which the gut microbiota interacts with the host is by means of metabolites. Microbial metabolites affect immune maturation, immune homeostasis, host energy metabolism and mucosal integrity maintenance (27). It has been reported that intestinal microbes selectively activate mucosal ECs and mesenchymal cells to promote specific angiogenic responses in a TLR- and Nod-like receptor-dependent manner (28). An array of studies have elucidated that microbial metabolites can regulate tumor angiogenesis. Butyrate, derived from the microbiota, promotes angiogenesis (29). Nicotinic acid effectively resists iodoacetamide-induced colitis by improving pathological angiogenesis and inflammation in a GPR109A-dependent manner (30). Another study demonstrated that a polypeptide of *Escherichia coli* and its tripeptide analogs promoted tumor cell invasion and angiogenesis, thus potentially affecting tumor metastasis (31). Dysregulation of the intestinal flora leads to increased intestinal permeability and chronic inflammation, resulting in increased production of IL-6, IL-1B, TNF-A, and VEGF-A, which ultimately intensifies pathological angiogenesis (32).

Overall, accumulating evidence has implicated the angiogenic process in various microbiota-associated human diseases. Herein, we investigated diabetic angiogenesis influenced by the microbiome, aiming to provide a broad understanding of how angiogenesis is involved in the diabetic state and how the microbiome regulates angiogenesis.

Tryptophan is an indispensable amino acid that can be taken up only *via* the diet. Tryptophan metabolism in different tissues is related to various physiological functions (33). A tryptophan metabolite of the gut microbiota, 3-IAId acid, attenuates inflammation (34). The potential benefits provided by indole derivatives have been highlighted in animal models of colitis and experimental autoimmune encephalomyelitis (35, 36). Moreover, 3-IAId has been found to promote angiogenesis in HUVECs (5). Our observations are consistent with previous studies showing that 3-IAId has proangiogenic properties. A study also showed that 3-IAld could restore epithelial barrier integrity and function, in turn ameliorating the metabolic complications (glucose tolerance and obesity) associated with the intake of a HFD (37). 3-IAId also significantly attenuated skin inflammation in mice with MC903-induced AD-like dermatitis, and this effect was blocked by an AHR antagonist and abolished in AHR-null mice (34). Remarkably, a recent metabolomic study showed that an increased fecal concentration of 3-IAld was associated with resistance to radiation-induced pathology, leading to hematopoietic, gastrointestinal, and cerebrovascular injuries (38). This finding points to a biological function of endogenous 3-IAld and suggests that its administration may provide long-term radioprotection. Studies also indicate that the therapeutic activity of 3-IAld, appropriately delivered through targeted pharmaceutics, may encompass metabolic and organ inflammatory pathology in murine models. Of interest, a pantissue AHR signature has recently highlighted 3-IAld among the metabolites downstream of the L-amino acid oxidase catabolism of Trp that were associated with AHR-driven cancer cell motility and immunosuppression (39). These studies qualify 3-IAld as a potent agent capable of strengthening epithelial barrier function, immune homeostasis and colonization resistance at mucosal and likely distal sites (40). Corresponding with previous studies, our study also demonstrated the protective role of 3-IAId in diabetic limb perfusion.

In conclusion, we have demonstrated that tolerance against severe limb ischemia under hyperglycemia is at least in part attributable to microbial metabolites and that supplementation with 3-IAId is sufficient to improve neoangiogenesis caused by limb ischemia in diabetic mice. We have also shown that this angiogenic response is dependent on mitochondrial function. Therefore, 3-IAId could be an attractive molecule for treating PAD in patients with diabetic vascular complications.

## Limitations

The present study has several limitations. Here, we first confirmed the protective role of 3-IAId in diabetic angiogenesis, as evidenced by improved EC tube formation, alleviated cell apoptosis, reduced ROS production and mitigated mitochondrial dysfunction. However, the potential mechanisms are still elusive and need further study. Additionally, male mice were used in our experiments for the sake of less hormone variability. The outcome in female mice may not be identical to these findings. Furthermore, the effect of 3-IAld on control animals remains unclear and needs to be addressed in future studies. Nevertheless, the findings demonstrate an essential role of 3-IAId in diabetic angiogenesis.

## Data Availability Statement

The original contributions presented in the study are included in the article/supplementary material, further inquiries can be directed to the corresponding authors.

## Ethics Statement

The animal study was reviewed and approved by Animal Protection and Utilization Committee of the Shanxi Cardiovascular Hospital.

## Author Contributions

XM, FW, and JA conceived and designed the study. XM, JY, GY, LL, XH, and GW performed the animal and cell culture experiments. XM, FW, JY, and JA interpreted the data and wrote the manuscript. JA and FW supervised the study and reviewed and edited the manuscript. All authors approved the final manuscript.

## Conflict of Interest

The authors declare that the research was conducted in the absence of any commercial or financial relationships that could be construed as a potential conflict of interest.

## Publisher’s Note

All claims expressed in this article are solely those of the authors and do not necessarily represent those of their affiliated organizations, or those of the publisher, the editors and the reviewers. Any product that may be evaluated in this article, or claim that may be made by its manufacturer, is not guaranteed or endorsed by the publisher.
